# Strength in numbers? The fragility index of studies from the Scandinavian knee ligament registries

**DOI:** 10.1007/s00167-019-05551-x

**Published:** 2019-06-12

**Authors:** Eleonor Svantesson, Eric Hamrin Senorski, Adam Danielsson, David Sundemo, Olof Westin, Olufemi R. Ayeni, Kristian Samuelsson

**Affiliations:** 1 Department of Orthopedics, Institute of Clinical Sciences, The Sahlgrenska Academy, University of Gothenburg, Gothenburg, Sweden; 2 Department of Health and Rehabilitation, Institute of Neuroscience and Physiology, The Sahlgrenska Academy, University of Gothenburg, Gothenburg, Sweden; 3 Department of Orthopedics, Sahlgrenska University Hospital, Mölndal, Sweden; 4 Division of Orthopaedic Surgery, Department of Surgery, McMaster University, Hamilton, ON Canada

**Keywords:** Registry, ACL, Anterior cruciate ligament, Fragility, Statistics, Revision, Contralateral, Laxity

## Abstract

**Purpose:**

The fragility index (FI) is a metric to evaluate the robustness of statistically significant results. It describes the number of patients who would need to change from a non-event to an event to change a result from significant to non-significant. This systematic survey aimed to evaluate the feasibility of applying the FI to findings related to anterior cruciate ligament (ACL) reconstruction in the Scandinavian knee ligament registries.

**Methods:**

The PubMed, EMBASE, Cochrane Library and AMED databases were searched. Studies from the Scandinavian knee ligament registers were eligible if they reported a statistically significant result (*p* < 0.05) for any of the following dichotomous outcomes; ACL revision, contralateral ACL reconstruction or the presence of postoperative knee laxity. Only studies with a two-arm comparative analysis were included. Eligibility assessment, data extraction and quality assessment were performed by two independent reviewers. The dichotomous analyses were stratified according to the grouping variable for the two comparative arms as follows; age, patient sex, activity at injury, graft choice, drilling technique, graft fixation, single- versus double-bundle, concomitant cartilage injury and country. The two-sided Fisher’s exact test was used to calculate the FI of all statistically significant analyses.

**Results:**

From 158 identified studies, 13 studies were included. They reported statistical significance for a total of 56 dichotomous analyses, of which all but two had been determined by a time-to-event analysis. The median sample size for the arms was 5540 (range 92–38,666). The mean FI for all 56 dichotomous analyses was 80.6 (median 34.5), which means that a mean of 80.6 patients were needed to change outcome status to generate a non-significant result instead of a significant one. Seventeen analyses (30.4%) immediately became non-significant when performing the two-sided Fisher’s exact test and, therefore, had an FI of 0. The analyses related to age were the most robust, with a mean FI of 178.5 (median 116, range 1–1089). The mean FI of the other grouping variables ranged from 0.5 to 48.0.

**Conclusion:**

There was large variability in the FI in analyses from the Scandinavian knee ligament registries and almost one third of the analyses had an FI of zero. The FI is a rough measurement of robustness when applied to registry studies, however, future studies are needed to determine the most appropriate metric for robustness in registry studies. The use of the FI can provide clinicians with a deeper understanding of significant study results and promotes an evidence-based approach in the clinical care of patients.

**Level of evidence:**

Systematic review of prospective cohort studies, Level II.

**Electronic supplementary material:**

The online version of this article (10.1007/s00167-019-05551-x) contains supplementary material, which is available to authorized users.

## Introduction

A large number of studies related to anterior cruciate ligament (ACL) reconstruction have been published from the Scandinavian knee ligament registries over the past decade [1, 2]. Many of these studies have aimed to determine predictors and risk factors for an additional ACL reconstruction, i.e. a revision or a contralateral ACL reconstruction, or used the presence of postoperative laxity as a measurement of primary ACL reconstruction failure [2]. A *P* value of less than 0.05 or a 95% confidence interval (CI) excluding the defined null value have been used as the threshold of significance when drawing conclusions on statistically significant predictors. Meeting these criteria implies that the null hypothesis, stating that there would be no difference in outcome depending on the investigated predictor, has been rejected, meaning that the predictor is likely to have a true effect on the outcome after ACL reconstruction.

The concept of a *P* value was first described by Sir Ronald Fisher and aids in the interpretation of a given result [3]. Although Fisher never did set a threshold for significance, a *P* value of less than 0.05 shows that a result that is similar to or more extreme than that observed would be found in fewer than 5% of repeated tests, on condition that the null hypothesis was true. It is therefore commonly accepted that a level of significance of 5% is sufficient to conclude that the observed result has not occurred by chance. Nonetheless, the *P* value says nothing about the robustness of an analysis and the interpretation of *P* values is many times misunderstood by researchers [4–6]. The fragility index (FI) was developed to evaluate the robustness of significant findings in randomised controlled trials (RCTs). More specifically, the FI describes the minimum number of patients in the group with the fewest events that would need to change from a non-event to an event to change the result from significant to non-significant [7]. Although the FI has not previously been applied to registry studies, it should follow the same principle. For example, there are studies from the Scandinavian knee ligament registries reporting that the use of hamstring tendon (HT) autograft significantly increases the risk of ACL revision compared with the use of patellar tendon (PT) autograft [8–10]. The FI for these studies would describe how many patients in the PT group would need to change from not undergoing an ACL revision to undergoing one to change the analysis to non-significant. The FI is thus a measurement of the number of events (e.g., ACL revisions) on which the statistical significance depends. In other words; the lower the FI, the more fragile the result.

Recently, the FI was evaluated for 48 RCTs in sports medicine and arthroscopic surgery [11]. Worryingly, the median FI of the included studies was two [11], meaning that drawing conclusions in current clinical trials of sports medicine is in fact based on the outcome of a very limited number of patients. One of the main methodological strengths of the Scandinavian registries is the prospective data collection from a large population. In fact, the registries together comprise data from over 70,000 primary ACL reconstructions [12]. Large study samples increase the robustness of a statistical analysis, however, the FI of the statistically significant findings presented from the Scandinavian registries has not been evaluated. This is important knowledge since it allows for a more precise interpretation of the results and promotes an evidence-based approach in the clinical care of patients. The purpose of this systematic survey was to evaluate the applicability of the FI to registry studies by determining the FI of all analyses from the Scandinavian registries related to any of the following dichotomised outcomes; ACL revision, contralateral ACL reconstruction and the presence of postoperative residual knee laxity.

## Materials and methods

### Eligibility criteria

Original studies written in English from the Danish, Norwegian and Swedish knee ligament registries were eligible for inclusion if they reported statistically significant results for any of the following dichotomous outcomes; ACL revision, contralateral ACL reconstruction, or the presence of residual knee laxity after ACL reconstruction. A statistically significant result was defined as a *P* value of < 0.05 or a 95% CI excluding a null value, under the null hypothesis that there would be no difference between groups. For ratio calculations, such as relative risk calculations, odds ratios or hazard ratio calculations, the definition of significance was a 95% CI excluding one. Only studies comparing a dichotomised outcome between two study groups were included, including studies using a dichotomised time-to-event analysis. Studies were excluded if information needed to calculate the FI was missing, e.g., data on the number/proportion of patients in each group, or the number/proportion of events in each group. For studies where only a proportion (%) was presented, the number of patients or the number of events was calculated for each group. Additionally, studies including data from registries outside Scandinavia were excluded.

### Literature search

The literature search was performed by an expert in electronic search methods at the Sahlgrenska University Hospital library on 9 May 2017. An updated literature search was performed on 20 April 2018. The searched databases were the PubMed, EMBASE, the Cochrane Library and AMED electronic databases. Search terms were mapped to relevant MeSH terms or subject headings where possible. Three concepts were used to enter search terms into the databases: Concept 1—‘Register’, ‘registry’, ‘registers’, and ‘registries’. Concept 2—‘Sweden’, ‘Swedish’, ‘Denmark’, ‘Danish’, ‘Norway’, ‘Norwegian’, ‘Scandinavia’, ‘Scandinavian’ and ‘Nordic countries’. Concept 3—‘Anterior cruciate ligament’, ‘Anterior cruciate ligament injuries’, ‘Anterior cruciate ligament reconstruction’, ‘Posterior cruciate ligament’ and ‘Posterior cruciate ligament reconstruction’. The ‘OR’ operator was used to group the keywords in each concept. Subsequently, the results from each concept were combined with the ‘AND’ operator. In addition, an e-mail was sent to the registry holder of each Scandinavian registry with a request for a list of publications from the registry. Two authors independently screened all abstracts and full texts, where needed, to identify eligible studies.

### Data extraction

Data were extracted independently by two authors using an electronic piloted form (Microsoft Excel, Microsoft Corp; Version 1812). The following data were extracted for each included study; total sample size, specification of the dichotomous outcome measurement, specification of the grouping variable, number of patients in each group, number of patients experiencing an event (the outcome) in each group, the unadjusted and adjusted (where applicable) statistically significant *P* value or 95% CI and information on the statistical analysis. All statistically significant results from a study originating from a dichotomous analysis by comparing two groups were extracted. So, if a study performed more than one two-group comparison for a dichotomous outcome, data for each analysis were extracted. If there was any disagreement, it was resolved by consulting a third author.

### Outcome

The dichotomised outcomes considered for this review were additional ACL reconstruction (either revision or contralateral ACL reconstruction) or postoperative knee joint laxity (yes/no). The dichotomous evaluation of postoperative knee joint laxity was defined according to the definition used in the original studies, i.e. positive pivot shift test (yes/no) and increased anteroposterior laxity of > 2 mm compared with the healthy knee (yes/no).

### Quality assessment

A standardised method for assessing internal validity (bias) in registry studies is lacking. The Downs and Black checklist for randomised and non-randomised studies primarily assesses the reporting quality of studies [13] and was determined to be the best available tool for quality assessment in this study. The checklist originally comprised 27 items scored on a 0–2 scale, yielding a maximum score of 30 points. Items number 14, 15, 23 and 24 are related to randomisation and were, therefore, excluded due to not being applicable to the included studies. Similarly, item 27 (power analysis) and item 21 could not be applied to the included studies. Item 21 was excluded as all the studies aiming to analyse two or more registries would score zero (patients not recruited from the same population), even though the quality of the multi-registry studies could be high. Therefore, a modified checklist yielding a maximum score of 22 points was used. Each study was assessed independently by two authors.

### Statistical analysis

The FI was calculated using two-by-two tables, according to the method described by Walsh et al. [7]. The *P* values for the extracted original data were first recalculated by applying a two-sided Fisher’s exact test. If the result was still significant (*p *< 0.05), the FI was calculated by adding the number of events to the group with the fewest number of events (or lowest risk of event/outcome), while subtracting the same number from the non-events in the group to keep the group sample size constant. Events were added until the *P* value of the two-sided Fisher’s exact test was no longer significant (*p* ≥ 0.05). The smallest number of patients that were required to change from a non-event to an event to obtain a *p* ≥ 0.05 was defined as the FI. All calculations were performed using Microsoft Excel (Microsoft Corp; Version 1812) and SPSS version 25 (IBM Corp; 2017).

The common methodology for reporting the FI for RCTs has been to report only one FI per study, by limiting the FI calculation to only the primary outcome measurement or the first statistically significant result presented in the study abstract [7, 11]. Registry studies often perform multiple analyses for a dichotomous outcome. For example, the dichotomous outcome of ACL revision may be compared between two age groups, two ACL graft choices and two graft fixation devices in the same study. The FI was, therefore, calculated for all the statistically significant results in each study and the results were organised and reported according to the specific predictor studied, i.e., the grouping variable. The grouping variables were classified as either patient- or surgery-related and reported under separate subheadings for readability purposes. Additionally, a subanalysis for the mean and median FI was performed after excluding analyses with an FI of zero. An FI of zero is thought to describe a highly fragile significance, as it means that zero patients need to change from a non-event to an event in order not to obtain significance when applying Fisher’s exact test to the analysis. However, as most studies from the Scandinavian knee ligament registries originally used statistics other than Fisher’s exact test [1, 2], there is a risk that using Fisher’s exact test might underestimate the FI. An FI of zero would be the most extreme underestimation and the subanalysis was therefore performed to compare the overall FI with and without analyses with an FI of zero.

## Results

### Study selection

**Fig. 1:**
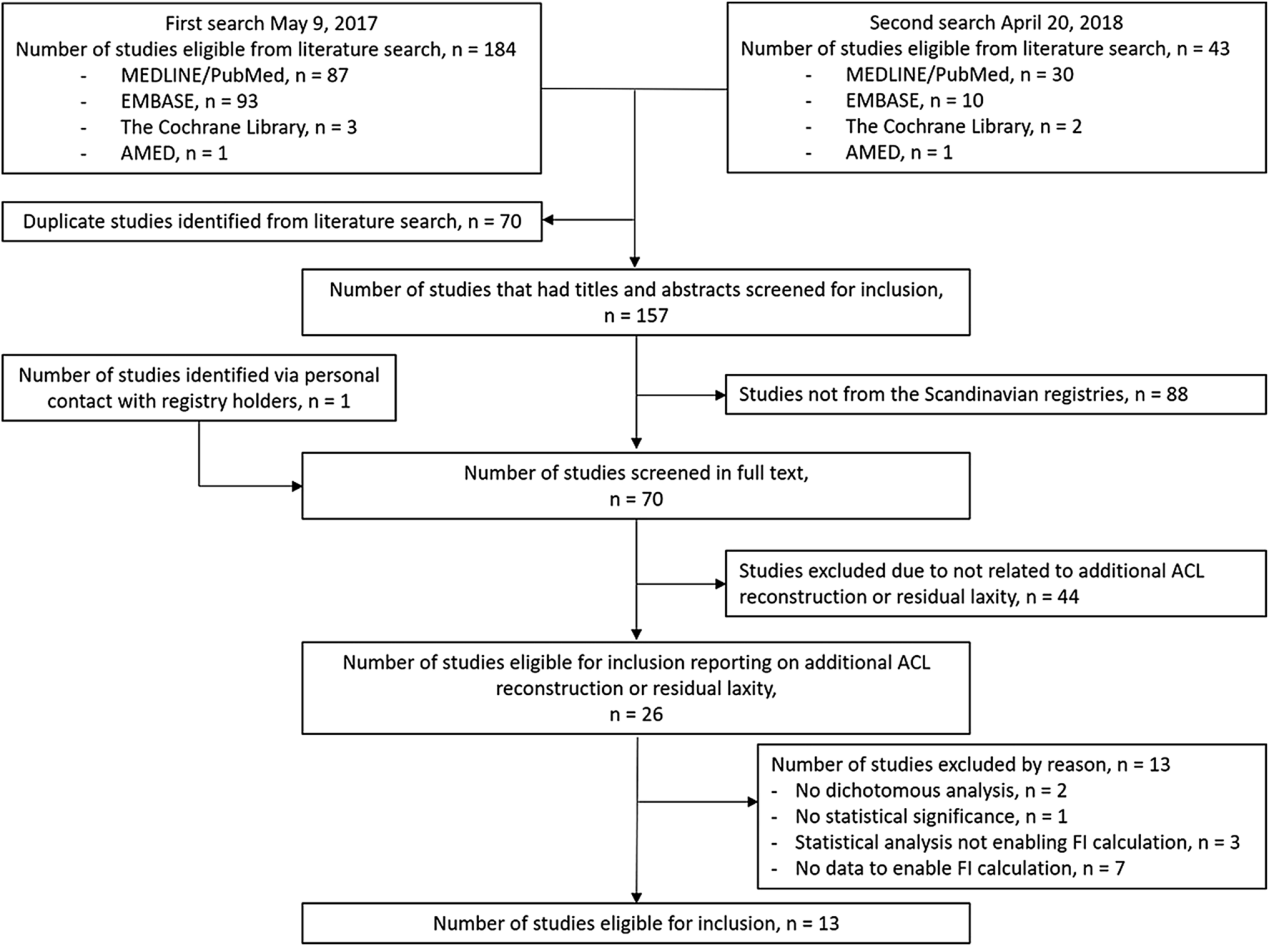
The study selection process. *FI* fragility index

The literature search yielded a total of 157 studies assessed for eligibility and one additional study was identified via communication with a registry holder. After a full-text review, 26 studies originating solely from the Scandinavian registries remained, which also reported on additional ACL reconstruction or postoperative residual knee laxity. Of these, two studies were excluded, as they did not perform any dichotomised statistical test [14, 15], one study was excluded due to not reporting any statistically significant result [16] and three studies were excluded due to applying a statistical analysis that did not enable FI calculation [17–19]. The remaining 20 studies reported at least one dichotomous outcome with statistical significance and a statistical test that enabled the calculation of FI. However, seven of these studies were excluded on the basis of not reporting the data needed for the calculation of the FI [20–26]. Finally, 13 studies were included for further analysis. The study selection process is presented in Fig. 1 and Online Appendix 1 presents the reason for excluding the studies that reported on additional ACL reconstruction or residual knee laxity.

### Overall study characteristics

The 13 included studies had a total of 56 separate dichotomous analyses, of which 49 analyses determined the outcome of ACL revision, three determined the outcome of a contralateral ACL reconstruction, three determined the outcome of residual knee laxity one year postoperatively and one analysis determined the outcome of either an ACL revision or a contralateral ACL reconstruction. The following variables were identified as determining the groups in the studies; age [8, 27–31], patient sex [30], activity at the time of injury [8, 32], HT versus PT autograft [8–10], femoral drilling technique [28, 33], graft fixation technique [27, 34, 35], single- versus double-bundle ACL reconstruction [27, 36], concomitant cartilage injury [8, 28, 30] and country where the ACL reconstruction was performed [27]. The Downs and Black score ranged from 13 to 18, with a median score of 17 of a maximum possible score of 22 (Table 1).

**Table 1:** Quality appraisal of included studies according to the Downs and Black checklist

Author	Journal	Hypothesis described	Main outcome described	Patient characteristics described	Interventions described	Principal confounders stated	Main findings described	Estimates of outcome variability	Adverse events reported	Characteristics of patients lost to F/U	Actual probability values	Subject asked to participate representative
		1	2	3	4	5	6	7	8	9	10	11
Aga (2017)	CORR	1	1	1	1	2	1	1	0	0	1	1
Ahldén (2012)	AJSM	1	1	1	1	0	1	1	0	1	1	1
Desai (2017)	KSSTA	1	1	1	1	1	1	1	0	1	1	1
Fauno (2014)	OJSM	1	1	1	1	2	1	1	0	1	0	1
Gifstad (2014)	AJSM	1	1	1	1	1	1	1	0	1	1	1
Persson (2014)	AJSM	1	1	1	1	2	1	1	0	1	1	1
Persson (2015)	AJSM	1	1	1	1	2	1	1	0	1	1	1
Persson (2018)	Acta Orthop	0	1	0	0	2	1	1	0	0	1	1
Rahr-Wagner (2014)	AJSM	1	1	0	1	2	1	1	0	0	0	1
Rahr-Wagner (2013)	Arthroscopy	1	1	0	1	2	1	1	0	0	1	1
Snæbjörnsson (2017)	KSSTA	1	1	1	1	1	1	1	0	0	1	1
Soreide (2016)	AJSM	1	1	1	1	2	0	1	0	1	1	1
Svantesson (2016)	KSSTA	1	1	1	1	1	1	1	0	0	1	1

Author	Subjects prepared to participate representative	Staff/facilities representative of regular treatment	Results based on data dredging made clear	Different length of follow-up adjusted	Appropriate statistics	Compliance reliable	Outcome valid and reliable	Recruitment over same period	Adjustment for confounders	Loss to F/U taken into account	Total
	12	13	16	17	18	19	20	22	25	26	
Aga (2017)	0	1	1	1	1	0	1	1	1	0	17
Ahldén (2012)	0	1	1	1	1	0	1	1	0	0	15
Desai (2017)	0	1	1	1	1	0	1	1	1	0	17
Fauno (2014)	0	1	1	1	1	0	1	1	1	1	18
Gifstad (2014)	1	0	1	1	1	0	1	1	1	1	18
Persson (2014)	0	1	1	1	1	0	1	1	1	0	18
Persson (2015)	0	1	0	1	1	0	1	1	1	0	17
Persson (2018)	0	1	0	1	1	0	1	1	1	0	13
Rahr-Wagner (2014)	0	1	1	1	1	0	1	1	1	0	15
Rahr-Wagner (2013)	0	1	1	1	1	0	1	1	1	0	16
Snæbjörnsson (2017)	0	1	1	1	1	0	1	1	1	0	16
Soreide (2016)	0	1	1	1	1	0	1	1	1	0	17
Svantesson (2016)	0	1	1	1	1	0	1	1	0	1	16

*Acta Orthop* acta orthopaedica, *AJSM* American journal of sports medicine, *CORR* clinical orthopaedics and related research, *KSSTA* knee surgery, sport traumatology, arthroscopy, *OJSM* orthopaedic journal of sports medicine

The median sample size for the arms was 5540 (range 92–38,666). The median in sample size difference between the two arms was 5464.5 patients (range 26–31,930). The median number of events for the outcome of additional ACL reconstruction (ACL revision or contralateral ACL reconstruction) was 159 (range 9–1171) and for residual laxity 264 (range 195–729). The mean FI for all 56 dichotomous analyses was 80.6, while the median was 34.5. Seventeen analyses (30.4%) became non-significant when performing the two-sided Fisher’s exact test to their contingency table and had therefore an FI of 0. All the analyses are summarised in Tables 2 and 3.

**Table 2:** The fragility index of patient-related factors for dichotomous events

Grouping variable	Author (year)	Dichotomous event	Arm 1	Arm 2	Sample size arm 1	Sample size arm 2	Events arm 1	Events arm 2	*p* value or 95% CI (unadjusted/adjusted*)	Statistical test	Fragility index	Mean fragility index	Median fragility index
Age	Aga et al. (2017)	Revision	Age 14–19	Age 20–24	14,733	12,645	985	568	< 0.001*	Cox regression	204	178.5	116.0
		Revision	Age 14–19	Age 25–29	14,733	9280	985	252	< 0.001*	Cox regression	309		
		Revision	Age 14–19	Age 30–60	14,733	24,120	985	403	< 0.001*	Cox regression	1089		
	Desai et al. (2017)	Revision	Age 13–15	Age 36–49	1300	3350	74	36	< 0.001	Kaplan–Meier	108		
		Revision	Age 16–20	Age 36–49	5075	3350	252	36	< 0.001	Kaplan–Meier	100		
		Revision	Age 21–25	Age 36–49	3667	3350	117	36	< 0.001	Kaplan–Meier	45		
		Revision	Age 26–30	Age 36–49	2513	3350	43	36	0.040	Kaplan–Meier	1		
		Revision	Age 13–25	Age 26–49	10,042	7640	443	109	< 0.001	Kaplan–Meier	183		
	Fauno et al. (2014)	Revision	Age 13–15	Age > 20	327	11,496	22	233	3.48 (2.24–5.38)/3.23 (2.05–5.08)*	Cox regression	263		
		Revision	Age 15–20	Age > 20	2888	11,496	140	233	2.57 (2.09–3.18)/2.50 (2.01–3.11)*	Cox regression	230		
	Gifstad et al. (2014)	Revision	Age 15–19	Age 20–24	10,947	8518	480	286	0.77 (0.67–0.90)/0.78 (0.67–0.90)*	Cox regression	39		
		Revision	Age 15–19	Age 25–29	10,947	6702	480	145	0.47 (0.39–0.57)/0.47 (0.39–0.57)*	Cox regression	108		
		Revision	Age 15–19	Age 30–34	10,947	5471	480	83	0.32 (0.25–0.40)/0.31 (0.25–0.40)*	Cox regression	121		
		Revision	Age 15–19	Age 35–39	10,947	5093	480	70	0.29 (0.23–0.37)/0.28 (0.22–0.37)*	Cox regression	119		
		Revision	Age 15–19	Age 40–44	10,947	4073	480	48	0.25 (0.19–0.34)/0.25 (0.19–0.34)*	Cox regression	101		
		Revision	Age 15–19	Age ≥ 45	10,947	3400	480	30	0.20 (0.14–0.28)/0.19 (0.13–0.28)*	Cox regression	93		
	Snaebjornsson et al. (2017)	Contralateral ACLR	Age 13–15	Age 36–49	1300	3350	88	45	0.002	Kaplan–Meier	132		
	Soreide et al. (2016)	Revision	Age 15–19	Age > 29	1680	3166	106^a^	41^a^	0.001*	Cox regression	116		
		Revision	Age 20–29	Age > 29	2647	3166	82^a^	41^a^	0.001*	Cox regression	31		
Patient sex	Snaebjornsson et al. (2017)	Contralateral ACLR	Female	Male	7669	10,013	266	260	0.001	Kaplan–Meier	35	–	–
Activity at injury	Ahlden et al. (2012)	Revision/contralateral ACLR	Female football players 15–18y	Male football players 15–18y	118	92	26^a^	9^a^	0.02	X2 test	2	16.0	5.5
	Gifstad et al. (2014)	Revision	Football	Alpine activities	18,810	6083	527	110	0.65 (0.53–0.79)/0.65 (0.53–0.79)*	Cox regression	32		
		Revision	Football	Other sports	18,810	12,103	527	285	0.83 (0.72–0.96)/0.85 (0.73–0.98)*	Cox regression	9		
		Revision	Football	Other/unknown	18,810	1033	527	19	0.57 (0.36–0.90)/0.54 (0.34–0.86)*	Cox regression	0		
		Revision	Handball	Football	5260	18,810	191	527	1.28 (1.08–1.51)/1.23 (1.04–1.45)*	Cox regression	53		
		Revision	Traffic/work	Football	2113	18,810	66	527	1.07 (0.83–1.38)/1.44 (1.12–1.87)*	Cox regression	0		

Arm 1 has the highest risk of an event across all analyses

^a^Number has been calculated from a proportion (%)

*Adjusted *P* value

*ACLR* anterior cruciate ligament reconstruction, *CI* confidence interval, *y* years

**Table 3:** The fragility index of surgery-related factors for dichotomous events

Grouping variable	Author (year)	Dichotomous event	Arm 1	Arm 2	Sample size arm 1	Sample size arm 2	Events arm 1	Events arm 2	*p* value or 95% CI (unadjusted/adjusted*)	Statistical test	Fragility index	Mean fragility index	Median fragility index
HT vs PT	Gifstad et al. (2014)	Revision	HT graft	PT graft	38,666	6736	1042	156	< 0.001	Log-rank test	0	15.0	10.0
	Persson et al. (2014)	Revision	HT graft	PT graft	9215	3428	362	69	< 0.001*	Cox regression	40		
	Rahr Wagner et al. (2014)	Revision	HT graft	PT graft	11,676	1971	312	47	1.50 (1.11–2.04)/1.41 (1.03–1.92)*	Cox regression	0		
		1y positive pivot shift	PT graft	HT graft	1025	4554	195^a^	729^a^	0.81 (0.68–0.96)*	Logistic regression	20		
Femoral drilling technique	Desai et al. (2017)	Revision	TP reference	TT non-anatomic	6685	1296	162	40	0.049/0.041*	Cox regression	0	48.0	17.0
		Revision	TP anatomic	TP reference	4036	6685	146	162	0.028/0.018*	Cox regression	34		
		Revision	TP drilling	TT drilling	12,440	5110	380	167	< 0.001/< 0.001*	Cox regression	0		
	Rahr-Wagner et al. (2013)	Revision	AM drilling	TT drilling	1945	6430	39	102	2.01 (1.39–2.92)/2.04 (1.39–2.99)*	Cox regression	0		
		1y positive pivot shift	AM drilling	TT drilling	1056	2949	206^a^	401^a^	2.86 (2.40–3.41)*	Cox regression	95		
		1y sagittal laxity > 2 mm	AM drilling	TT drilling	1051	2807	208^a^	320^a^	3.70 (2.09–4.43)*	Cox regression	159		
Graft fixation	Aga et al. (2017)	Revision	Femoral metal screw	Femoral others (not bioscrew or button)	9788	25,089	357	902	<0.001*	Cox regression	0	37.4	1.0
		Revision	Femoral metal screw	Femoral button	9788	24,872	357	906	0.01*	Cox regression	0		
		Revision	Tibial bioscrew	Tibial metal screw	10,859	17,564	399	609	0.02*	Cox regression	0		
		Revision	Tibial others (not bioscrew or button)	Tibial metal screw	30,873	17,564	1171	609	0.04*	Cox regression	0		
	Persson et al. (2015)	Revision	HT endobutton/RCI screw	PT	2339	3806	72	24	< 0.001*	Cox regression	62		
		Revision	HT EzLoc/WasherLoc	PT	1352	3806	29	24	< 0.001*	Cox regression	27		
		Revision	HT Endobutton/Biosure HA	PT	1209	3806	49	24	< 0.001*	Cox regression	87		
		Revision	HT Endobutton/Intrafix	PT	687	3806	23	24	< 0.001*	Cox regression	55		
		Revision	HT TransFix II/metal interference screw	PT	620	3806	9	24	0.047*	Cox regression	2		
	Persson et al. (2018)	Revision	Femoral Endobutton	Femoral Rigidfix	14,106	12,041	342	316	0,7 (0.6–0.8)/0,7 (0.6–0.8)*	Cox regression	0		
		Revision	Femoral Endobutton	Femoral Transfix	14,106	3652	342	100	0.7 (0.5–0.8)/0.7 (0.6–0.9)*	Cox regression	0		
		Revision	Tibial retro interference screw	Tibial interference screw	508	18,640	27	462	1.8 (1.2–2.6)/1.9 (1.3–2.9)*	Cox regression	216		
SB vs DB	Aga et al. (2017)	Revision	DB HT graft	SB PT graft	994	7790	37	219	0.01*	Cox regression	0	0.5	0
	Svantesson et al. (2017)	Revision	SB	DB	21,846	614	689	12	0.01/0.019*	Cox regression	0		
		Revision	SB TP reference	DB	5609	614	146	12	0.015/0.037*	Cox regression	0		
		Revision	SB TP anatomic	DB	3449	614	133	12	0001/0002*	Cox regression	2		
Concomitant cartilage injury	Desai et al. (2017)	Revision	No cartilage injury	Cartilage injury	13,084	4598	435	117	0.002	Kaplan–Meier	9	19.7	9.0
	Gifstad et al. (2014)	Revision	No cartilage injury	Cartilage injury	35,618	9784	1007	191	< 0.001	Log-rank test	50		
	Snaebjornsson et al. (2017)	Contralateral ACLR	No cartilage injury	Cartilage injury	13,084	4598	408	118	0.01	Kaplan–Meier	0		
Country	Aga et al. (2017)	Revision	Norway	Sweden	14,648	26,299	613	868	< 0.001*	Cox regression	130	–	–

Arm 1 has the highest risk of an event across all analyses

^a^Number has been calculated from a proportion (%)

*Adjusted *P* value

*ACLR* anterior cruciate ligament reconstruction, *AM* anteromedial, *CI* confidence interval, *DB* double-bundle, *HT* hamstring tendon autograft, *PT* patellar tendon autograft, *SB* single-bundle, *TP* transportal; *TT* transtibial, *y* years

### Patient-related factors

The following variables were identified as patient-related; age [8, 27–31], patient sex [30] and activity at time of injury [8, 32]. A total of 19 two-arm analyses for age (18 related to the outcome of ACL revision and one to contralateral ACL reconstruction) were identified. The FI for age ranged from 1 to 1089, with a mean FI of 178.5 and a median of 116.0. The analysis on patient sex as a factor for contralateral ACL reconstruction had an FI of 35.0. There were six analyses on activity at time of injury (five related to the outcome of ACL revision and one to either ACL revision or a contralateral ACL reconstruction). The FI for these analyses ranged from 0 to 53, with a mean FI of 16.0 and a median of 5.5. All analyses with a patient-related factor as the grouping variable are summarised in Table 2.

### Surgery-related factors

The following variables were identified as surgery-related; HT versus PT autograft [8–10], femoral drilling technique [28, 33], graft fixation [27, 34, 35], single- versus double-bundle ACL reconstruction [27, 36], concomitant cartilage injury [8, 28, 30] and country where the ACL reconstruction was performed [27]. With regard to HT versus PT autograft, three analyses were related to the outcome of ACL revision and one analysis to the outcome of a positive pivot shift one year postoperatively. The FI ranged from 0 to 40, with a mean FI of 15.0 and median of 10.0. The drilling technique comparisons were made between transtibial drilling and the anteromedial or transportal drilling technique (four analyses related to the outcome of ACL revision, one to the outcome of a positive pivot shift test at one year postoperatively and one to the outcome of > 2 mm sagittal laxity at one year postoperatively). The FI ranged from 0 to 159, with a mean FI of 48.0 and a median of 17.0. Graft fixation was investigated in 12 two-arm analyses. The FI ranged from 0 to 216, with a mean FI of 37.4 and a median of 1.0. Single- versus double-bundle was investigated in four analyses, with an FI ranging from 0 to 2 (mean FI 0.5 and median FI 0). With regard to concomitant cartilage injury, there were two analyses related to the outcome of ACL revision and one to contralateral ACL reconstruction. The FI ranged from 0 to 50, with a mean FI of 19.7 and a median of 9.0. With regard to country where the ACL reconstruction was performed, one analysis related to the outcome of ACL revision. The FI of the significant difference between the countries was 130. All analyses with a surgery-related factor as the grouping variable are summarised in Table 3.

### Subanalysis

When excluding the 17 analyses with an FI of 0, a total of 39 analyses remained. The FI of those analyses ranged from 1 to 1089, with a mean FI of 115.7 and a median FI of 87.0 (data not shown).

## Discussion

This most important finding of this study was that the FI varied substantially across dichotomous analyses from the Scandinavian knee ligament registries. Although almost one third of the analyses had an FI of zero, the analyses related to age generally had the most robust FI, with a mean FI of 178.5 (range 1–1089). In fact, the majority of the analyses had a higher FI than what previously has been reported from RCTs related to orthopaedic surgery [11, 37]. However, the variable FI underlines that there are difficulties in the interpretation of robustness in analyses from these registry studies.

The FI has previously been applied exclusively to RCTs. A median FI of 2 (IQR 1–3) was reported when assessed in 40 RCTs related to orthopaedic spine surgery [37]. Similarly, a median FI of 2 (IQR 1–2.8) was found in 48 RCTs related to arthroscopy and sports medicine surgery [11]. It was concluded that the statistical significance in current orthopaedic RCTs is fragile and that relatively small sample sizes and few outcome events are contributory factors [37]. The large study samples provided by registry studies could theoretically increase the robustness of significant findings. On the other hand, it is not known whether the use of the FI is feasible for registry studies, as there are some fundamental discrepancies in the study design compared with RCTs. A well-designed RCT is thought to exclude confounding factors by assuming an equal distribution of both measured and unmeasured factors due to randomisation and blinding. Registry studies are instead susceptible to confounders and bias, which is commonly dealt with by statistical adjustments. The FI is calculated independently of whether or not the tested *P* value originates from an adjusted analysis, which is important, as most analyses included in this study were adjusted. Moreover, despite the fact that the FI has previously been applied to time-to-event outcomes in RCTs [7, 11], time-to-event outcomes are more common in registry studies. Time-to-event is not considered when calculating the FI, which means that the FI becomes a rougher measurement for these analyses.

It is obvious that the FI had greater variability in registry studies compared with previous studies of RCTs [11, 37]. The variability of the FI questions the feasibility of using this metric on registry studies, especially since a strong contributor to the variable FI probably is the heterogeneous data analyses. For example, there is no consensus on how to stratify age groups in the Scandinavian knee ligament registry studies. This aggravates a comparison of the FI across studies, since the FI in addition to describe the actual robustness also will be affected by group size and age difference between groups. Moreover, almost one third of the analyses had an FI of zero, which is difficult to interpret for analyses which originally used statistics other than Fisher’s exact test and found significance. Does an FI of zero indicate fragility or is it the result of applying a statistical test that was not deemed to be the most appropriate test in the original study? Interestingly, Walsh et al. [7] applied the FI to 399 trials published in high-impact journals and found that 70% of the trials with an FI of zero originally were analysed using time-to-event analysis [7]. This indicates that time-to-event analyses are particularly susceptible to an FI of zero, which might explain the large proportion of analyses with an FI of zero in this study. Not surprisingly, the mean and median FI were considerably higher when the analyses with an FI of zero were excluded in the subanalysis. Further research is needed to determine the most appropriate methodology for FI calculation in studies using time-to-event analysis. Until then, it could be argued that time-to-event analyses with an FI of zero should be excluded to not severely skew the overall FI.

To draw conclusions regarding the feasibility of using the FI on registry studies, the FI perhaps needs to be assessed in a larger number of registry studies, which could strengthen the data and narrow the range of the FI. That could potentially also enable a determination of the most robust predictors for ACL failure. In this study, most predictors included analyses from only one or two studies. The inclusion of few analyses per predictor makes the FI analysis sensitive to outliers and makes the process of determining the most robust predictor vulnerable. One should however bear in mind that only significant analyses are considered for the FI calculation and few included analyses for a predictor could mean that the reported significance is an exception among several non-significant findings. Patient sex could be used to exemplify this, where only one analysis was included with an FI of 35. Although the FI for patient sex is difficult to interpret based on a single analysis, it should be remembered that seven other studies using ACL revision as the outcome and three other studies using contralateral ACL reconstruction as the outcome found non-significant results when comparing patient sex in the Scandinavian registries [2]. The interpretation of the FI must therefore also be set in the context of the cumulative evidence.

Not all predictors were, however, limited by few analyses, and the feasibility of using the FI is strengthened when determining the FI for the two predictors with most analyses (age with 19 analyses and graft fixation with 12 analyses), as the result reflects previous literature on the subject. The literature is unanimous when it comes to young age as a risk factor for an additional ACL reconstruction [38–42]. In agreement with this, the FI for age was by far the highest, which indicates that the FI calculation is able to provide a reliable estimate of robustness. This is further emphasised by the higher FI in analyses with an increasing age difference between the compared groups. With regard to graft fixation, six of 12 analyses had an FI of 0 and the mean FI for all the analyses was 37.4. For this reason, graft fixation does not predict an additional ACL reconstruction with the same certainty as age, which is supported by the contradictory literature regarding the impact of graft fixation [43–45].

In the light of the limitations associated with using the FI on registry studies, it is our opinion that the FI could be regarded as a rough measurement of robustness for registry studies and that it could be used to compare confidence in the results across analyses with a similar statistical methodology. There is reason to believe that the FI could be a valuable method for registry studies, especially for those using statistical methods that are perfectly compatible with the FI, such as Chi-square or Fisher’s exact test. The large amount of data comprised in registries should intuitively increase the robustness, but it is important to find a metric to quantify this objectively in registry studies. Although there might be outcomes in registry studies that are more or less appropriate for the use of FI, researchers should be encouraged to calculate and report the FI whenever possible. The strength in numbers of registry studies does not compensate for other limitations, such as confounders, bias and an inability to provide causality. Care must be taken not to overestimate the effect of a higher FI in registry studies compared with RCTs, as RCTs still remain the gold standard to determine the efficacy of an intervention. This study is also limited by the fact that analyses from seven studies needed to be excluded, as data on study arm size and the number of events were not reported. Future studies should preferably report these numbers. It should also be mentioned that the outcome of additional ACL reconstruction may underestimate the true rate of failed ACL reconstructions. This becomes especially relevant to consider in studies with small FIs, since the robustness of significance in these circumstances is likely to be even more vulnerable to “hidden” failures not proceeding to an ACL revision.

## Conclusion

There was large variability in the FI in analyses from the Scandinavian knee ligament registries and almost one third of the analyses had an FI of zero. The FI is a rough measurement of robustness when applied to registry studies, however, future studies are needed to determine the most appropriate metric for robustness in registry studies. The use of the FI can provide clinicians with a deeper understanding of significant study results and promotes an evidence-based approach in the clinical care of patients.

## Electronic supplementary material

Below is the link to the electronic supplementary material.

Supplementary material 1 (DOCX 15 kb)
