# Supplementary material for: Strength in numbers? The fragility index of studies from the Scandinavian knee ligament registries
Source: Knee Surg Sports Traumatol Arthrosc. 2019 Jun 12;28(2):339–52. doi: 10.1007/s00167-019-05551-x (PMC6995986; doi:10.1007/s00167-019-05551-x)
Supplement: Supplementary file 1 — Supplementary material 1 (DOCX 15 kb) [file 167_2019_5551_MOESM1_ESM.docx]

| **Appendix 1. Studies excluded by reason for exclusion** | | | |
| --- | --- | --- | --- |
| Author | Study | Publication year | Reason for exclusion |
| Andernord et al. | Surgical Predictors of Early Revision Surgery After Anterior Cruciate Ligament Reconstruction: Results From the Swedish National Knee Ligament Register on 13,102 Patients | 2014 | Did not report data to enable analysis |
| Andernord et al. | Predictors of contralateral anterior cruciate ligament reconstruction: a cohort study of 9061 patients with 5-year follow-up | 2015 | Did not report data to enable analysis |
| Fältström et al. | Predictors for additional anterior cruciate ligament reconstruction: data from the Swedish national ACL register | 2016 | Did not report data to enable analysis |
| Granan et al. | Associations between inadequate knee function detected by KOOS and prospective graft failure in an anterior cruciate ligament-reconstructed knee | 2015 | Did not report data to enable analysis |
| Kvist et al. | Results from the Swedish national anterior cruciate ligament register | 2014 | Did not report data to enable analysis |
| Lind et al. | Incidence and outcome after revision anterior cruciate ligament reconstruction: results from the Danish registry for knee ligament reconstructions | 2012 | Did not report data to enable analysis |
| Rahr-Wagner et al. | Comorbidities in Patients With Anterior Cruciate Ligament Reconstruction Compared With Matched Controls Without Anterior Cruciate Ligament Injury From Danish Registries | 2015 | Did not report data to enable analysis |
| Lind et al. | The first results from the Danish ACL reconstruction registry: epidemiologic and 2 year follow-up results from 5,818 knee ligament reconstructions | 2009 | No dichotomous outcome |
| Rahr-Wagner et al. | Validation of 14,500 operated knees registered in the Danish Knee Ligament Reconstruction Register: registration completeness and validity of key variables | 2013 | No dichotomous outcome |
| Eysturoy et al. | The Influence of Graft Fixation Methods on Revision Rates After Primary Anterior Cruciate Ligament Reconstruction | 2018 | Statistical analysis not enabling FI calculation |
| Snaebjornsson et al. | Graft Diameter as a Predictor for Revision Anterior Cruciate Ligament Reconstruction and KOOS and EQ-5D Values: A Cohort Study From the Swedish National Knee Ligament Register Based on 2240 Patients | 2017 | Statistical analysis not enabling FI calculation |
| Andernord et al. | Patient predictors of early revision surgery after anterior cruciate ligament reconstruction: a cohort study of 16,930 patients with 2-year follow-up | 2015 | Statistical analysis not enabling FI calculation |
| Bjornsson et al. | No difference in revision rates between single- and double-bundle anterior cruciate ligament reconstruction: a comparative study of 16,791 patients from the Swedish national knee ligament register | 2015 | No statistical significance |
